# Effect of Combined Stressors on C. elegans Lifespan

**DOI:** 10.1093/geroni/igab046.2505

**Published:** 2021-12-17

**Authors:** Bradford Hull, George Sutphin

**Affiliations:** 1 University of Arizona, Arizona, United States; 2 The University of Arizona, Tucson, Arizona, United States

## Abstract

Cellular stress is a fundamental component of age-associated disease. Cells experience many forms of stress (oxidative, heavy metal, etc.), and as we age the burden of stress and resulting damage increases while our cells’ ability to deal with the consequences becomes diminished due to dysregulation of cellular stress response pathways. By understanding how cells respond to stress we aim to slow age-associated deterioration and develop treatment targets for age-associated disease. The majority of past work has focused on understanding responses to individual stressors. In contrast, how pathology and stress responses differ in the presence of multiple stressors is relatively unknown; we investigate that here. We cultured worms on agar plates with different combinations of arsenic, copper, and DTT (which create oxidative/proteotoxic, heavy metal, and endoplasmic reticulum (ER) stress, respectively) at doses that result in 20% lifespan reduction individually and measured the effect on lifespan. We found that arsenic/copper and arsenic/DTT combinations created additive lifespan reductions while the copper/DTT combination created an antagonistic lifespan reduction when compared to controls (p<0.05). This antagonistic toxicity suggests an interaction either between the mechanisms of toxicity or the cellular response to copper and DTT. We are now evaluating the impact of copper and DTT individually and in combination on unfolded protein and heavy metal response pathways to understand the underlying mechanism of the interaction. Additionally, we are continuing to screen stressors to identify combinations that cause non-additive (synergistic or antagonistic) toxicity to build a comprehensive model of the genetic stress response network in C. elegans.

